# Predicting microbe–disease association based on graph autoencoder and inductive matrix completion with multi-similarities fusion

**DOI:** 10.3389/fmicb.2024.1438942

**Published:** 2024-09-06

**Authors:** Kai Shi, Kai Huang, Lin Li, Qiaohui Liu, Yi Zhang, Huilin Zheng

**Affiliations:** ^1^College of Computer Science and Engineering, Guilin University of Technology, Guilin, China; ^2^Guangxi Key Laboratory of Embedded Technology and Intelligent Systems, Guilin University of Technology, Guilin, China

**Keywords:** microbe–disease associations, network similarities, similarity fusion, inductive matrix completion, graph autoencoder

## Abstract

**Background:**

Clinical studies have demonstrated that microbes play a crucial role in human health and disease. The identification of microbe-disease interactions can provide insights into the pathogenesis and promote the diagnosis, treatment, and prevention of disease. Although a large number of computational methods are designed to screen novel microbe-disease associations, the accurate and efficient methods are still lacking due to data inconsistence, underutilization of prior information, and model performance.

**Methods:**

In this study, we proposed an improved deep learning-based framework, named GIMMDA, to identify latent microbe-disease associations, which is based on graph autoencoder and inductive matrix completion. By co-training the information from microbe and disease space, the new representations of microbes and diseases are used to reconstruct microbe-disease association in the end-to-end framework. In particular, a similarity fusion strategy is conducted to improve prediction performance.

**Results:**

The experimental results show that the performance of GIMMDA is competitive with that of existing state-of-the-art methods on 3 datasets (i.e., HMDAD, Disbiome, and multiMDA). In particular, it performs best with the area under the receiver operating characteristic curve (AUC) of 0.9735, 0.9156, 0.9396 on abovementioned 3 datasets, respectively. And the result also confirms that different similarity fusions can improve the prediction performance. Furthermore, case studies on two diseases, i.e., asthma and obesity, validate the effectiveness and reliability of our proposed model.

**Conclusion:**

The proposed GIMMDA model show a strong capability in predicting microbe-disease associations. We expect that GPUDMDA will help identify potential microbe-related diseases in the future.

## 1 Introduction

Microbes, often known as microorganisms, are tiny or ultramicroscopic organisms that include bacteria, fungi, eukaryotes, archaea, and other microorganisms (Ley, 2022). Microbes inhabiting human skin, saliva, oral mucosa, and the gastrointestinal tract are important for human health and life (Aggarwal et al., 2023). Many studies have shown that the abnormality in microbial communities can lead to diseases, such as intestinal autoimmune diseases, multiple sclerosis, diabetes, neurological disorders, and cancer (Miyauchi et al., 2023; Loh et al., 2024; White and Sears, 2024). However, it is difficult to identify the relationship between microbes and diseases based on conventional biological experiments since they are time-consuming, labor-intensive, and expensive. Therefore, it is necessary to develop computational approaches with high accuracy and efficiency to identify latent microbe–disease associations.

Nowadays, many methods have been developed to screen the microbe–disease interactions (Wen et al., 2021; Wang L. et al., 2022). Network-based methods usually utilize the topological information of different networks to identify potential new relationships. These methods with fewer parameters are relatively simple and easy to understand, but the prediction accuracy is influenced by the available associations and cannot be applied to new diseases or microbes without any known association in the network. The initial work is that Chen et al. (2017) developed the first computational model (KATZHMDA) for predicting microbe–disease association based on the network measurement Katz by integrating the number and length of walks between nodes. Yan et al. (2020) designed a prediction method based on a bi-random walk on the heterogeneous network including the microbe network, diseases network, and known microbe–disease associations network. In addition, other methods, considering network projection, label propagation, multi-similarity fusion, etc., are developed to discover potential microbe–disease interactions (Qu et al., 2019; Luo and Long, 2020; Yin et al., 2022a,b).

Matrix factorization is another kind of method to screen potential associations of microbe–disease entities, which maps the high-dimensional matrix into the product of low-dimensional matrices (Wang et al., 2019; Ma et al., 2020; Peng et al., 2020; Liu Y. et al., 2021; Liu et al., 2023). Although it solved the problem of data sparsity well, there were also several limitations, such as poor interpretability and slow training speed. For example, Ma et al. (2020) presented a computational model (MDNMF) based on non-negative matrix factorization to deduce latent disease-related microbe candidates. Peng et al. (2020) adopted positive-unlabeled learning and random walk to select negative samples and then utilized logistic matrix factorization with neighborhood regularization to find possible microbe–disease associations. Liu et al. (2023) used low-rank representation to obtain structural similarity information and utilized collaborative filtering to identify unknown microbe–disease pairs.

In addition, traditional machine learning methods can achieve an accurate prediction of future data, which starts from some training samples. The prediction accuracy of machine learning methods is trustworthy, but the problem of optimal values of model parameters is still unsolved. Furthermore, it is a challenge for them to obtain appropriate feature representations for microbes and diseases including all key information from the similarity network and association matrix (Wang et al., 2017; Peng et al., 2018; Ding et al., 2021; Xu et al., 2021). Peng et al. (2018) developed an adaptive Boosting enhanced microbe–disease association (ABHMDA) prediction model, the core of which was to calculate the association probability of microbe–disease pairs by combining weak classifiers into strong classifiers. Xu et al. (2021) used the Kronecker regularized least squares to calculate prediction scores with different Kronecker similarities. Moreover, some researchers applied graph representation learning to achieve rich feature representations for microbe–disease association prediction (Lei and Wang, 2020; Wang Y. et al., 2022; Yueyue et al., 2022). One representative work is that Yueyue et al. (2022) used an embedding algorithm called GraRep to learn global graph features on the heterogeneous network and adopted a support vector machine classifier to obtain the probability score of the microbe–disease associations.

Recently, deep learning approaches have become popular solutions for predicting microbe–disease associations. The basic concept is to use multiple processing layers to automatically learn the representation of data and multiple levels of abstraction. As deep learning is widely used in different fields and shows satisfactory performance, many deep learning-based prediction methods (such as graph convolutional networks and graph attention networks) have been developed for microbe–disease association prediction (Li et al., 2021; Long et al., 2021; Chen and Lei, 2022; Peng et al., 2023; Zhu et al., 2023). For example, to conclude the underlying microbe–disease associations, the novel back-propagation neural network model (BPNNHMD) was designed (Li et al., 2021). Long et al. (2021) integrated a graph attention network with induction matrix completion to predict possible microbial candidates for diseases. Peng et al. (2023) proposed a multi-view feature aggregation model to identify microbe–disease interactions, in which both linear and nonlinear features were extracted, respectively. Moreover, Chen and Lei (2022) employed metapath to aggregate graph neural networks for finding potential microbe–disease pairs on a microbe–drug–disease heterogeneous network.

Although the existing methods have attained significant progress in microbe–disease association prediction, there are still some limitations. First, most methods only use a single dataset, so the accuracy and reliability of the data need to be further improved. Second, in the microbe–disease network, plenty of edges focus on a handful of disease nodes, predicting potential edges easily biased toward those with more known associations with disease or microbe. Third, most methods cannot be applied to the prediction of a new disease or new microbe (i.e., disease or microbe without any known associations) owing to similarities depending on known microbe–disease association. Additionally, the calculated similarity often contains noise due to the lack of high-quality experimental data, which affects the accuracy of prediction. Finally, some methods cannot accurately capture the complex nonlinear associations.

To address these challenges, we proposed an improved deep learning framework with graph autoencoders and inductive matrix completion (GIMMDA) to identify the latent microbe–disease associations. First, we constructed a robust microbe–disease heterogeneous network, and the disease network and microbe network are constructed by a similarity fusion strategy on different similarities including functional similarity and topological similarity. Then, we adopted an end-to-end framework to integrate graph autoencoder and inductive matrix completion, where the information from microbe and disease space are co-trained. Finally, the score matrix constructed from node representations of graph autoencoders is utilized to predict the potential microbe–disease interactions. Our model can be considered an end-to-end model that directly maps input data to the final output, automatically extracting features from raw data and making predictions by probability. In the 5-fold cross-validation (5-fold CV), our model obtained a reliable performance.

## 2 Materials and methods

### 2.1 The known human microbe–disease associations

Currently, the microbe–disease pairs prediction mainly depends on several popular databases, including HMDAD (Ma et al., 2017), Disbiome (Janssens et al., 2018), gutMDisorder (Cheng et al., 2020), MicroPhenoDB (Yao et al., 2020), MASI (Zeng et al., 2021) and Peryton (Skoufos et al., 2021). The interactions are screened from biomedical articles with manual curation or text-mining, which is shown in Table 1. However, the robust datasets are still lacking, due to different adopt strategies, noise introduction, and update absence. For more reliable prediction, we selected two datasets HMDAD (450 associations between 39 diseases and 292 microbes) and Disbiome (8,645 associations between 351 diseases and 1,582 microbes) to validate the performance of the model. Furthermore, we constructed a new dataset (multiMDA) including 1,124 associations between 287 diseases and 567 microbes, where those associations are found in at least two of the six datasets mentioned above, after unifying the disease name and microbe taxonomy.

**Table 1:** Summaries of the human microbe-associated datasets.

Databases	Associations	Microbe	Disease
HMDAD (Ma et al., 2017)	450	292	39
Disbiome (Janssens et al., 2018)	8,645	1,582	351
gutMDisorder (Cheng et al., 2020)	1,187	409	81
MicroPhenoDB (Yao et al., 2020)	5,500	1773	496
MASI (Zeng et al., 2021)	629	123	56
Peryton (Skoufos et al., 2021)	1747	469	38
multiMDA	1,124	567	287

In this study, an adjacent matrix $A\in {\mathbb{R}}^{n_{d}\times n_{m}}$ with 0–1 entries is constructed to represent the known microbe–disease associations, where $A\left(d_{i},m_{j}\right)=1$ if a disease i is associated with a microbe j , otherwise $A\left(d_{i},m_{j}\right)=0$ .

### 2.2 Interaction profile similarity for disease and microbe

The interaction profile similarity is widely used to predict correlation between biological entities, and the underlying assumption is that similar diseases (microbes) generally have the same interaction patterns with similar microbes (diseases) and vice versa. In this study, several popular similarities based on the adjacency matrix A are adopted, including Gaussian Interaction Profile (GIP) kernel similarity, Cosine similarity, and Jaccard similarity.

#### 2.2.1 Gaussian interaction profile kernel similarity

Gaussian interaction profile kernel similarity is a kernel measuring the similarity of nodes in a network, in which the interaction profile of a node is a binary vector specifying the presence or absence of interaction with other nodes (van Laarhoven et al., 2011). In this study, we represent the interaction profile of the disease i as a binary vector to encode the interaction pattern with every microbe. It is the ith row of the adjacency matrix A , denoting as $A\left(d_{i},:\right)$ . Similarly, the jth column of matrix A , known as $A\left(:,m_{j}\right)$ , is the microbe interaction profile for a microbe j . The Gaussian interaction profile kernel similarity for disease pairs or microbe pairs can be calculated as follows by Equations 1, 2:


(1) $$GD\left(d_{i},d_{j}\right)=\exp\left(-\gamma_{d}\left\|A\left(d_{i},:\right)-A{\left(d_{j},:\right)}^{2}\right\|\right)$$

(2) $$\begin{array}{c} GM\left(m_{i},m_{j}\right)=\exp\left(-\gamma_{m}\left\|A\left(:,m_{i}\right)-A{\left(:,m_{j}\right)}^{2}\right\|\right) \end{array}$$

where $\gamma_{d}$ and $\gamma_{m}$ denote the normalized kernel bandwidth (Equations 3, 4).


(3) $$\begin{array}{c} \gamma_{d}=\frac{\gamma_{d}^{\prime }}{\frac{1}{n_{d}}\sum_{k=1}^{n_{d}}\left\|A{\left(d_{k},:\right)}^{2}\right\|} \end{array}$$

(4) $$\begin{array}{c} \gamma_{m}=\frac{\gamma_{m}^{\prime }}{\frac{1}{n_{m}}\sum_{k=1}^{n_{m}}\left\|A{\left(:,d_{k}\right)}^{2}\right\|} \end{array}$$

where $\gamma_{d}^{\prime }$ and $\gamma_{m}^{\prime }$ are the initial bandwidth parameters, which generally are set to 1.

#### 2.2.2 Cosine similarity

Commonly, cosine similarity is a popular metric used to measure the similarity of related entities, which considers the cosine angle between vector representations of entities in Euclidean space. Then, we, respectively, constructed cosine similarity score matrixes for diseases and microbes under the adjacency matrix A , where row vectors are the disease representation and column vectors are the microbe representation. Therefore, the similarity metrics are defined as follows by Equations 5, 6:


(5) $$\begin{array}{c} CD\left(d_{i},d_{j}\right)=\cos\left(A\left(d_{i},:\right),\;\;A\left(d_{j},:\right)\right)=\frac{A\left(d_{i},:\right)\cdot A\left(d_{j},:\right)}{\left\|A\left(d_{i},:\right)\right\|\times \left\|A\left(d_{j},:\right)\right\|} \end{array}$$

(6) $$\begin{array}{c} CM\left(m_{i},m_{j}\right)=\cos\left(A\left(:,m_{i}\right),\;A\left(:,m_{j}\right)\right)=\frac{A\left(:,m_{i}\right)\cdot A\left(:,m_{j}\right)}{\left\|A\left(:,m_{i}\right)\right\|\times \left\|A\left(:,m_{j}\right)\right\|} \end{array}$$

where $CD\left(d_{i},d_{j}\right)$ represents the cosine similarity between disease i and j , and similarly, $CM\left(m_{i},m_{j}\right)$ is the microbe cosine similarity between microbe i and j . Furthermore, we normalized the cosine similarity to the range 0 to 1.

#### 2.2.3 Jaccard similarity

Jaccard similarity is a common proximity measurement for characterizing the similarity between two sets of objects. We adopted the following formula to calculate the disease similarity between disease i and disease j by Equation 7:


(7) $$\begin{array}{c} JD\left(d_{i},d_{j}\right)=\frac{\left|D_{i}\cap D_{j}\right|}{\left|D_{i}\cup D_{j}\right|} \end{array}$$

where $D_{i}=\left\{d_{k}|A{\left(d_{i},:\right)}_{d_{k}}=1\right\}$ , $D_{j}=\left\{d_{k}|A{\left(d_{j},:\right)}_{d_{k}}=1\right\}$ . Similarly, following the metric, we derived the microbe Jaccard similarity matrix $JM\in {\mathbb{R}}^{n_{m}\times n_{m}}$ .

### 2.3 Biological function similarity for disease and microbe

In addition to network topological information, wide pieces of evidence available from different biological sources are applied to improve accuracy for predicting microbe–disease associations. For disease resources, we considered disease-related symptom data, disease semantic information, and disease-gene functional information and also measured the similarity between microbes by the evolutionary distance of nucleotide sequences of microbes.

#### 2.3.1 Disease symptom similarity

Zhou et al. (2014) constructed a comprehensive human symptom–disease network, which made it possible to find similar diseases from the perspective of disease symptoms. With representation learning, each disease is usually represented by a vector of symptoms, $d_{i}=\left(w_{i1},w_{i2},\ldots ,w_{in}\right)$ , where $w_{ij}$ characterizes the importance of symptom $f_{i}$ to disease j , ranging from 0 to 1. In clinical research, there is a common phenomenon that different diseases present trend to the prevalence of different symptoms. To highlight heterogeneity, the association strength between symptom $f_{i}$ and disease j is measured as follows by Equation 8:


(8) $$\begin{array}{c} w_{ij}=W_{ij}\log\frac{n_{d}}{n_{i}}, \end{array}$$

where $n_{d}$ is the number of diseases, and $n_{i}$ is the number of diseases with symptom $f_{i}$ . $W_{ij}$ denotes if symptom $f_{i}\;$ and disease j is co-occurrent. The symptom-based disease similarity between disease pairs can be calculated as follows by Equation 9:


(9) $$\begin{array}{c} SymD\left(d_{i},d_{j}\right)=\frac{\sum_{l}d_{il}d_{jl}}{\sqrt{\sum_{l}d_{il}^{2}}\sqrt{\sum_{l}d_{jl}^{2}}}, \end{array}$$

where $d_{i,}$ and $d_{j}$ donate the symptom vector of the disease i and j , respectively.

#### 2.3.2 Disease semantic similarity

Semantic similarity is an important way of similarity measurement, which is widely applied to predict association. Therefore, we adopted semantic similarity to calculate the similarity between diseases. First, we downloaded MeSH descriptors from the National Library of Medicine,^1^ and each disease is represented by a directed acyclic graph (DAG) structure based on MeSH descriptors. By defining a semantic value for each disease, we calculated the semantic similarity between the two diseases. The semantic similarity between 0 and 1 is transformed into the disease semantic similarity matrix SeD (Yin et al., 2022a). For disease $d_{i}$ , $DAG\left(d_{i}\right)=\left(d_{i},T\left(d_{i}\right),E\left(d_{i}\right)\right)$ , where T( $d_{i}$ ) denotes the nodes in $DAG\left(d_{i}\right)$ , and $E\left(d_{i}\right)$ refers to the edges in $DAG\left(d_{i}\right)$ . The semantic contribution value of disease $d_{i}\;$ to disease $d_{j}$ can be calculated as $C_{D}^{1}\left(d\right)=\max\left\{f\times C_{D}^{1}\left(d^{\prime }\right)|d^{\prime }\in children\;of\;d\right\},d\neq D$ . We can get the semantic value of the disease $d_{j}$ $V_{s}^{1}\left(d_{j}\right)=\underset{d\in T\left(d_{j}\right)}{\sum }C_{d_{j}}^{1}\left(d\right)$ and the semantic similarity $S^{1}\left(d_{i},d_{j}\right)=\frac{\sum_{t\in T\left(d_{i}\right)\cap T\left(d_{j}\right)}C_{d_{i}}^{1}\left(t\right)+C_{d_{j}}^{1}\left(t\right)}{V_{s}^{1}\left(d_{i}\right)+V_{s}^{1}\left(d_{j}\right)}$ .

In addition, the semantic contribution value of the disease $d_{i}$ to disease $d_{j}$ can be calculated as $C_{d_{j}}^{2}\left(d_{i}\right)=-\log\frac{the\;number\;of\;DAGs\;including\;d}{the\;number\;of\;diseases\;}$ and the semantic value of disease $d_{j}$ is presented as $V_{s}^{2}\left(d_{j}\right)=\underset{d\in T\left(d_{j}\right)}{\sum }C_{d_{j}}^{2}\left(d\right)$ . For disease $d_{i}$ and disease $d_{j}$ , the semantic similarity value is presented as $S^{2}\left(d_{i},d_{j}\right)=\frac{\sum_{t\in T\left(d_{i}\right)\cap T\left(d_{j}\right)}C_{d_{i}}^{2}\left(t\right)+C_{d_{j}}^{2}\left(t\right)}{V_{s}^{2}\left(d_{i}\right)+V_{s}^{2}\left(d_{j}\right)}$ . The final disease semantic similarity can be formulated as follows by Equation (10):


(10) $$\begin{array}{c} SeD\left(d_{i},d_{j}\right)=\left(S^{1}+S^{2}\right)/2 \end{array}$$

#### 2.3.3 Disease gene functional similarity

Furthermore, we computed the disease functional similarity with disease-related genes. The underlying assumption is that phenotypically similar diseases usually interact with similar genes. The interactions between genes are available from the HumanNet dataset (Kim et al., 2022), in which an associated log-likelihood score (LLS) is used to evaluate the possible functional linkage between gene pairs. Given diseases i and j , the functional similarity can be formulated as follows by Equation 11:


(11) $$\begin{array}{c} FunD\left(d_{i},d_{j}\right)=\frac{\sum_{1\leq i\leq m}FS_{G_{b}}\left(g_{ai}\right)+\sum_{1\leq j\leq n}FS_{G_{a}}\left(g_{bj}\right)}{m+n}, \end{array}$$

where $G_{a}=\left\{g_{a1},g_{a2},\ldots ,g_{am}\right\}$ and $G_{b}=\left\{g_{b1},g_{b2},\ldots ,g_{bn}\right\}$ denote gene sets of $d_{i}$ and $d_{j}$ , respectively. The functional association between a gene and a gene set is defined as follows by Equations 12, 13:


(12) $$\begin{array}{c} FS_{G_{b}}\left(g_{ai}\right)=\underset{1\leq j\leq n}{\max}\left(F\left(g_{ai},g_{bj}\right)\right) \end{array}$$

(13) $$\begin{array}{c} FS_{G_{a}}\left(g_{bj}\right)=\underset{1\leq i\leq m}{\max}\left(F\left(g_{ai},g_{bj}\right)\right), \end{array}$$

where $F\left(g_{ai},g_{bj}\right)$ denotes the functional similarity score between gene $g_{ai}$ and gene $g_{bj}$ with log-likelihood score formula (LLS) (Kim et al., 2022).

#### 2.3.4 Microbe sequence similarity and evolutionary distance similarity

In molecular biology, the genetic sequences of microorganisms usually are considered to determine their structure, function, and behavior. Sequence similarity can measure how closely two related sequences are at the molecular level. Techniques, such as sequence alignment and comparison, are used to determine the degree of similarity between the nucleotide or amino acid sequences of different microorganisms. Higher sequence similarity suggests a closer evolutionary relationship, while lower similarity may indicate a more distant evolutionary divergence. In this study, we downloaded 16S rRNA gene sequences of microbiota in datasets from NCBI and compared the targeted sequence against the rest sequences in turn by the Basic Local Alignment Search Tool (BLAST+)(Camacho et al., 2009). The similarity value can be estimated based on the consistency of nucleotide sequences, where the normalized microbe sequence similarity matrix MIS consists of the identity of the alignment Equation 14.


(14) $$\begin{array}{c} MIS\left(m_{i},m_{j}\right)=\frac{Id\left(m_{i},m_{j}\right)-\min.\left(Id\right)}{\max.\left(Id\right)-\min.\left(Id\right)}, \end{array}$$

where Id denotes a matrix about the identity of the alignment with size m × m.

Furthermore, we consider the microbe evolutionary distance under the *p*-distance model, which represents the number of nucleotide substitutions occurring between a pair of sequences. The evolutionary distance score is obtained by applying MEGA7(Tamura et al., 2021), and the normalized evolutionary distance similarity can be expressed as follows Equation 15:


(15) $$\begin{array}{c} MES\left(m_{i},m_{j}\right)=\frac{Ed\left(m_{i},m_{j}\right)-\min.\left(Ed\right)}{\max.\left(Ed\right)-\min.\left(Ed\right)}, \end{array}$$

where the $\;Ed\left(m_{i},m_{j}\right)$ is the evolutionary distance score between microbe i and j .

#### 2.3.5 Microbe similarity based on disease semantic

We followed the hypothesis that functionally similar microbes are implicated in similar diseases (Wang et al., 2023) and obtained the microbe similarity based on disease semantics. If microbes $m_{i}$ and $m_{j}$ refer to the disease sets $D_{i}$ and $\;D_{j}$ , respectively, the max similarity between a disease d' and disease set D is defined as follows Equation 16:


(16) $$\begin{array}{c} SIM\left(d^{\prime },D\right)=\underset{d\in D}{\max}\left(SeD\left(d^{\prime },d\right)\right), \end{array}$$

where $SeD\left(d^{\prime },d\right)$ is the disease semantic similarity between d' and d , the final similarity between microbe $\;m_{i}$ and $m_{j}$ is calculated as follows Equation 17:


(17) $$\begin{array}{c} W_{MFS}\left(m_{i},m_{j}\right)=\frac{\sum_{d\in D_{j}}SIM\left(d,D_{i}\right)+\sum_{d\in D_{i}}SIM\left(d,D_{j}\right)}{\left|D_{i}\right|+\left|D_{j}\right|}. \end{array}$$

### 2.4 Construction of microbe–disease heterogeneous network

It is well known that precisely predicting microbe–disease association underlying a robust microbe–disease heterogeneous network. However, most studies insufficiently consider the similarity network of the biological entities (disease or microbe) in terms of different attributes (interaction profile and biological function information), to scream potential microbe-disease pairs due to the diversity of biological data.

**Figure 1:**
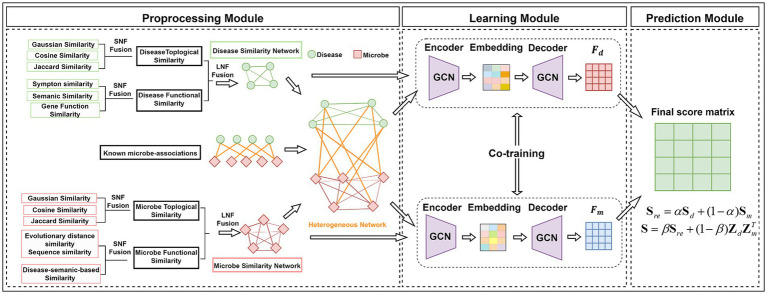
The flowchart of the GIMMDA model.

In this study, we constructed the similarity networks for biological entities, respectively, where each similarity network considers different fusion strategies including the network topology based on the interaction profile and the biological function as illustrated in Figure 1.

#### 2.4.1 Fusion strategy within the same attributes

For the same attributes (network topology based on interaction profile or biological function information), we adopted similarity network fusion (SNF) (Wang et al., 2014). SNF is a nonlinear combination method, which is used to fuse the similarity of the same biological entities from different similarity metrics.

In the SNF procedure, after defining the similarity matrix W of each view data, a normalized weight matrix P and a local affinity matrix S are constructed. Then, the fusion process based on message-passing theory starts from P as the initial state and S as the kernel matrix to iteratively update the similarity matrix on m datasets Equation 18:


(18) $$\begin{array}{c} P^{\left(v\right)}=S^{\left(v\right)}\times \frac{\sum_{k\neq v}P^{\left(k\right)}}{m-1}\times {\left(S^{\left(v\right)}\right)}^{T},v=1,2,\ldots m \end{array}$$

The final similarity matrix can be defined as by Equation 19:


(19) $$\begin{array}{c} P=\frac{\sum_{v=1}^{m}P^{\left(v\right)}}{m} \end{array}$$

For the disease entity, we derive the disease topological similarity matrix $DS_{top}$ by integrating three similarity metrics (GD, CD, and JD), and disease functional similarity matrix $DS_{fun}$ under considering similarity metrics (SymD, SeD, and FunD). In addition, microbe topological similarity $MS_{top}$ and microbe functional similarity matrix $MS_{fun}$ are obtained similarly.

#### 2.4.2 Fusion strategy between different attributes

Furthermore, the disease similarity $D_{s}$ and microbe similarity $M_{s}$ are integrated by linear fusion (LNF) as follows Equations 20, 21:


(20) $$\begin{array}{c} D_{s}=\mu DS_{top}+\left(1-\mu \right)DS_{fun} \end{array}$$

(21) $$\begin{array}{c} M_{s}=\mu MS_{top}+\left(1-\mu \right)MS_{fun}, \end{array}$$

where μ∈(0,1) is a weight parameter that controls topological similarity and functional similarity. Finally, the microbe–disease heterogeneous network is constructed based on similar networks Ds , Ms , and microbe–disease association A.

## 3 Method

In this study, we adopted an end-to-end framework GIMMDA to predict latent microbe–disease associations integrating the graph autoencoder and inductive matrix completion. The overview of the GIMMDA framework is shown in Figure 1. First, the preprocessing module integrates information from different biological sources using SNF and LNF methods. Second, the learning module learns new latent feature representations of microbes and diseases based on the graph autoencoder and inductive matrix completion. Finally, the prediction module utilizes the score matrix constructed from node representations of graph autoencoder to predict the potential microbe–disease interactions.

### 3.1 Graph autoencoder

Graph autoencoder is popularly applied to unsupervised learning and link prediction. Usually, graph autoencoder consists of the encoder and decoder, where the graph encoder generates an embedding matrix Z=f(X,Y) taking a feature matrix X and a graph adjacency matrix Y , and the decoder reconstructs the graph adjacency matrix $\hat{Y}=g\left(Z\right)$ or node features matrix $\hat{X}$ using pairs of node embedding vectors.

Here, we applied the graph autoencoder framework to obtain node representations on the disease network and microbe network, respectively, where the microbe–disease association matrix is regarded as the node attributes. First, we adopted graph convolution encoders to generate the node representation by aggregating node information from neighbors and graph structure information (Kipf and Welling, 2016) Equations 22, 23.


(22) $$\begin{array}{c} Z_{d}=\tanh\left(\hat{D_{s}}Relu\left(\hat{D_{s}}A^{T}\Psi_{d}^{\left(0\right)}\right)\Psi_{d}^{\left(1\right)}\right) \end{array}$$

(23) $$\begin{array}{c} Z_{m}=\tanh\left(\hat{M_{s}}Relu\left(\hat{M_{s}}A^{T}\Psi_{m}^{\left(0\right)}\right)\Psi_{m}^{\left(1\right)}\right), \end{array}$$

where $\Psi_{d}$ and $\Psi_{m}$ are the learnable weight parameters, and $\hat{D_{s}}$ and $\hat{M_{s}}$ denote the normalized adjacency matrix of the disease graph and microbe graph, respectively.

At the decoder part, we reconstruct the microbe–disease association matrix A with the embedding of the diseases and microbes Z_d_ and Z_m_, respectively, which can be expressed as follows Equations 24, 25:


(24) $$\begin{array}{c} S_{d}=sigmoid\left(\hat{D_{s}}Relu\left(\hat{D_{s}}Z_{d}\Theta_{d}^{\left(0\right)}\right)\Theta_{d}^{\left(1\right)}\right) \end{array}$$

(25) $$\begin{array}{c} S_{m}=sigmoid\left(\hat{M_{s}}Relu\left(\hat{M_{s}}Z_{m}\Theta_{m}^{\left(0\right)}\right)\Theta_{m}^{\left(1\right)}\right), \end{array}$$

where $\Theta_{d}$ and $\Theta_{m}$ are trainable parameter matrices.

In the graph autoencoder framework, we minimized the reconstruction loss of node features as follows by Equation 26:


(26) $$\begin{array}{c} Lr=\alpha {\left\|A-S_{d}\right\|}_{F}^{2}+\left(1-\alpha \right){\left\|A-S_{m}^{T}\right\|}_{F}^{2} \end{array}$$

where α∈(0,1) is the balance factor between the microbe and disease spaces.

### 3.2 Inductive matrix completion

Inductive matrix completion (IMC) usually is considered as transductive multi-label learning, where the association matrix can be generated by applying feature representations of its row and column entities to a low-rank matrix Z. In microbe–disease association prediction, the goal is to recover a feature projection matrix Z using the known microbe–disease association matrix A , which can be formulated as an optimization problem as follows by Equation 27 (Li et al., 2020):


(27) minΨd,Ψm‖A−ZdZmT‖F2+λ(‖Ψd‖F2+‖ΨmFF2‖),

where $Z_{m}\in {\mathbb{R}}^{n_{m}\times k}$ and $Z_{d}\in {\mathbb{R}}^{n_{d}\times k}$ are the new embedding representations of microbes and diseases, respectively. k denotes the embedding dimension, $\Psi_{d}$ and $\Psi_{m}$ are the learnable weight parameters. The inductive matrix completion loss can be defined as follows by Equation 28:


(28) $$\begin{array}{c} Lc={\left\|A-Z_{d}Z_{m}^{T}\right\|}_{F}^{2} \end{array}$$

### 3.3 Collaborative optimization

Minimizing Lr is equivalent to training graph autoencoders on microbe and disease networks, respectively. However, previous studies have shown that collaborative training can improve the accuracy of predicting associations of biological entities (Jin et al., 2021; Shi et al., 2021). We defined the total loss L of GIMMDA as follows by Equation 29:


(29) $$\begin{array}{c} \begin{array}{l} L=\underset{\Psi_{d},\Psi_{m},\Theta_{d},\Theta_{m}}{\min}\beta Lr+\left(1-\beta \right)Lc+\lambda \\ \;\;\;\;\left({\left\|\Psi_{d}\right\|}_{F}^{2}+{\left\|\Psi_{m}\right\|}_{F}^{2}+{\left\|\Theta_{d}\right\|}_{F}^{2}+{\left\|\Theta_{m}\right\|}_{F}^{2}\right). \end{array} \end{array}$$

Let $W=\left\{\Psi_{d}{\mathrm{,\Psi}}_{m}{\mathrm{,\Theta}}_{d}{\mathrm{,\Theta}}_{m}\right\}$ , the above equation can be rewritten as follows by Equation 30:


(30) $$\begin{array}{c} L=\underset{W}{\min}\beta Lr+\left(1-\beta \right)Lc+{\left\|\lambda W\right\|}_{F}^{2}, \end{array}$$

where β∈(0,1) is the balance factor between reconstruction loss and inductive matrix completion loss. The Adam (Jin et al., 2021) optimizer is used for optimization. Finally, the predicted score matrix S is obtained under the optimal model parameters follows by Equation 31.


(31) $$\begin{array}{c} S=\beta S_{re}+\left(1-\beta \right)Z_{d}Z_{m}^{T}, \end{array}$$

where $S_{re}=\alpha S_{d}+\left(1-\alpha \right)S_{m}$ .

The detailed steps of GIMMDA are summarized in Algorithm 1.

#### ALGORITHM 1. GIMMDA algorithm

**Table:** 

Input: initial interaction matrix A
Output: final score matrix S
1: Compute the disease similarity matrix $D_{s}$ and the microbe similarity matrix $M_{s}$
2: Compute the adjacent matrix of the disease graph $\hat{D_{s}}$ and microbe graph $\hat{M_{s}}$ respectively
3: repeat
4: Learn the embedding vectors of diseases and microbes via Encoder (expression here is defined in Equations 22, 23)
5: Reconstructed score matrix via Decoder (expression here is defined in Equations 24, 25)
6: Train the new feature representations of disease and microbe space through optimizing Loss and update W (i.e., parameters of graph convolutional networks) by Adam optimizer
7: Until Convergence
8: return $S=\beta S_{re}+\left(1-\beta \right)Z_{d}Z_{m}^{T}$

## 4 Results

### 4.1 Experiment setting

In our experiments, we performed a 5-fold CV on the association matrix under three different settings: a global test adopts randomly zeroed values to the association matrix; a horizontal test for diseases where rows of the association matrix are randomly zeroed; and a vertical test for microbes where columns of the association matrix are randomly zeroed.

The global test compares the ability to identify latent microbe–disease associations on all methods. Horizontal tests for diseases and vertical tests for microbes compare the ability to predict new diseases and microbes, respectively. To reduce the impact of random splitting on performance, we repeated the 5-fold CV of each method 10 times. The average AUC, F1 score, accuracy, sensitivity, and specificity values are used as the performance indicators.

### 4.2 Parameter selection

**Figure 2:**
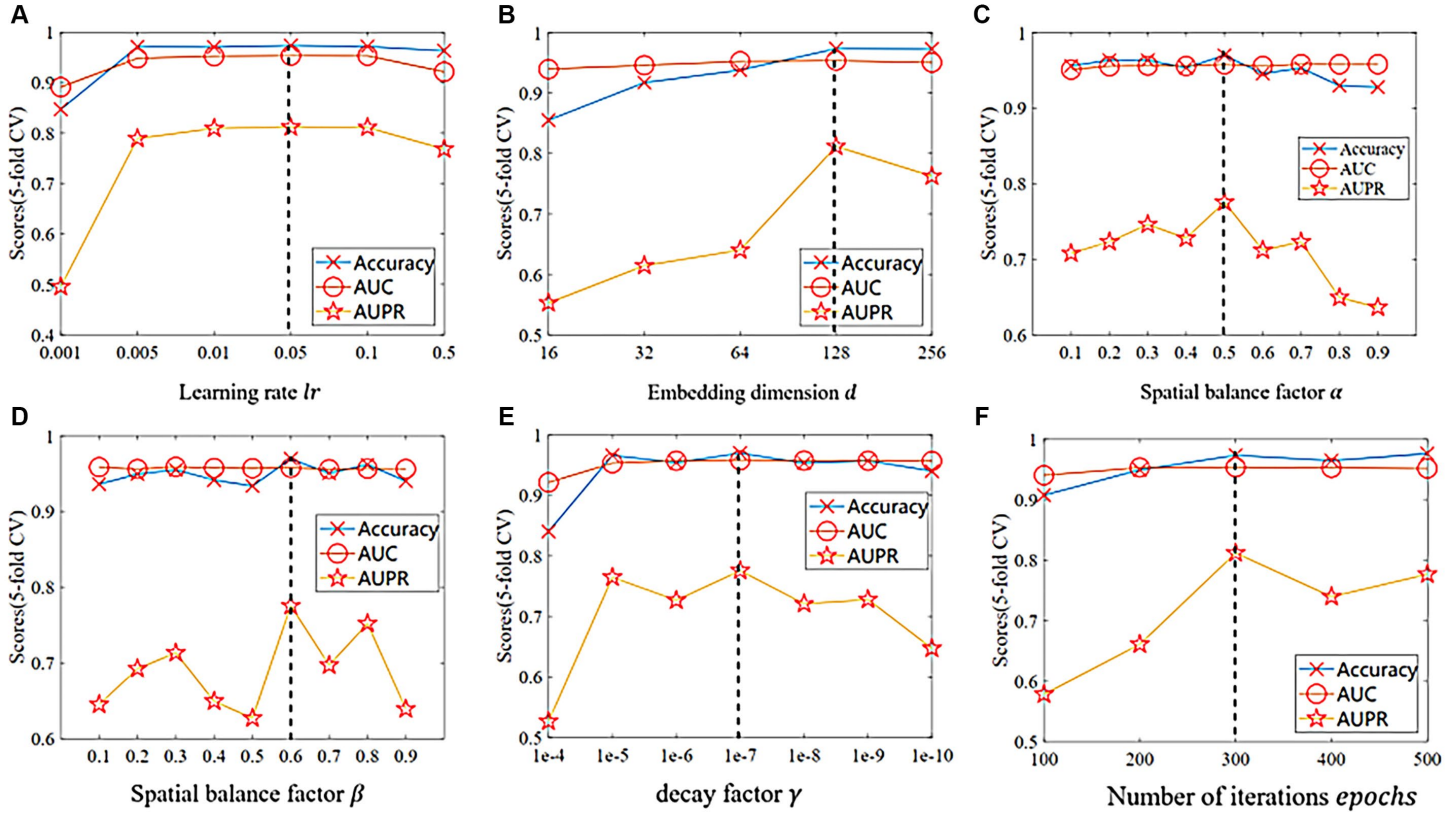
The comparison of different indicators in 5-fold CV on the multiMDA dataset for **(A)** learning rate lr , **(B)** embedding dimension d , **(C)** spatial balance factor α , **(D)** loss balance factor β , **(E)** decay factor λ , and **(F)** number of iterations epochs.

The proposed GIMMDA model involves six important hyperparameters, such as the learning rate lr , the dimension of the embedding k , the disease and microbe spatial balance factor α , the loss-term balance factor β , the decay factor of regularization γ, and the number of iterations epochs. It is worth noting that we used the global test in a 5-fold CV for parameter selection under the multiMDA dataset and considered different combinations of all parameters by grid search. As shown in Figure 2, our proposed model performs best when lr=0.05 , k=128 , α=0.5 , β=0.6 , $\lambda =10^{-7},$ and epochs=300.

### 4.3 Performance evaluation

In this study, we compared the proposed GIMMDA model with nine other state-of-the-art methods, including classical network-based methods [KATZHMDA (Chen et al., 2017), BRWMDA (Yan et al., 2020), and NTSHMDA (Luo and Long, 2020)], matrix factorization models [NBLPIHMDA (Wang et al., 2019) and MDLPHMDA (Qu et al., 2019)], and machine learning and deep learning models [LRLSHMDA (Wang et al., 2017), GATMDA (Long et al., 2021), BPNNHMDA (Li et al., 2021), and MVFA (Peng et al., 2023)].

Table 2 shows the performance comparison between GIMMDA and the other 9 methods in the global test of 5-fold CV based on HMDAD and Disbiome datasets. We observed that the AUC, F1 score, and accuracy values of the GIMMDA achieved 0.9735 ± 0.0050, 0.9140 ± 0.0239, and 0.9817 ± 0.0051 on the HMDAD dataset, which was the highest among all compared methods. Compared with the second-best method MVFA, the GIMMDA increased the AUC, F1 score, and accuracy by 2.17, 3.85, and 2.47%, respectively. On the Disbiome dataset, the GIMMDA also achieved the best performance in AUC, F1 score, accuracy, sensitivity, and specificity, which were 0.54, 7.5, 2.08, 12.31, and 9.36%, respectively, better than the second-best method MVFA.

**Table 2:** Performance comparison of the 10 methods under the global test of 5-fold CV on HMDAD and Disbiome datasets.

Datasets		Methods	AUC	F1 Score	Accuracy	Sensitivity	Specificity
HMDAD	Network-based	KATZHMDA	0.8331 ± 0.0171	0.8260 ± 0.0404	0.8578 ± 0.0541	0.7056 ± 0.0579	0.8591 ± 0.0523
		BRWMDA	0.8936 ± 0.0169	0.8884 ± 0.0386	0.8440 ± 0.0525	0.8013 ± 0.0628	0.8443 ± 0.0534
		NTSHMDA	0.8866 ± 0.0200	0.8868 ± 0.0428	0.8321 ± 0.0622	0.7991 ± 0.0678	0.8324 ± 0.0632
	Matrix factorization	NBLPIHMDA	0.9004 ± 0.0153	0.8949 ± 0.0247	0.8614 ± 0.0344	0.8107 ± 0.0407	0.8618 ± 0.0349
		MDLPHMDA	0.8942 ± 0.0186	0.8884 ± 0.0386	0.8249 ± 0.0418	0.8013 ± 0.0628	0.8443 ± 0.0534
	Machine learning	LRLSHMDA	0.8816 ± 0.0150	0.7964 ± 0.0322	0.7970 ± 0.0532	0.8138 ± 0.0527	0.7969 ± 0.0540
		GATMDA	0.9222 ± 0.0102	0.8772 ± 0.0277	0.8842 ± 0.0168	**0.9667 ± 0.0143**	0.8808 ± 0.0178
		BPNNHMDA	0.9242 ± 0.0103	0.8951 ± 0.0344	0.8371 ± 0.0616	0.8624 ± 0.0600	0.8369 ± 0.0626
		MVFA	0.9518 ± 0.0056	0.8755 ± 0.0707	0.9570 ± 0.0125	0.9004 ± 0.0181	0.9605 ± 0.0139
		GIMMDA	**0.9735 ± 0.0050**	**0.9140** ± **0.0239**	**0.9817 ± 0.0051**	0.8976 ± 0.0140	**0.9827 ± 0.0094**
Disbiome	Network-based	KATZHMDA	0.5340 ± 0.0060	0.4322 ± 0.0292	0.8575 ± 0.0233	0.2761 ± 0.0239	0.8594 ± 0.0224
		BRWMDA	0.8612 ± 0.0046	0.8702 ± 0.0233	0.7957 ± 0.0327	0.7709 ± 0.0363	0.7957 ± 0.0330
		NTSHMDA	0.8300 ± 0.0043	0.9065 ± 0.0105	0.6905 ± 0.0202	0.8292 ± 0.0175	0.6901 ± 0.0203
	Matrix factorization	NBLPIHMDA	0.8844 ± 0.0034	0.9033 ± 0.0104	0.7949 ± 0.0177	0.8238 ± 0.0173	0.7948 ± 0.0178
		MDLPHMDA	0.8889 ± 0.0026	0.9124 ± 0.0105	0.7935 ± 0.0165	0.8390 ± 0.0177	0.7934 ± 0.0166
	Machine learning	LRLSHMDA	0.7948 ± 0.0034	0.9444 ± 0.0076	0.5949 ± 0.0132	0.8947 ± 0.0137	0.5940 ± 0.0133
		GATMDA	0.8431 ± 0.0081	0.8015 ± 0.0188	0.8115 ± 0.0168	0.8849 ± 0.0133	0.8013 ± 0.0189
		BPNNHMDA	0.7771 ± 0.0149	0.8443 ± 0.0603	0.6912 ± 0.0861	0.7349 ± 0.0906	0.6912 ± 0.0861
		MVFA	0.9102 ± 0.0027	0.8728 ± 0.0037	0.9010 ± 0.0146	0.7861 ± 0.0059	0.8284 ± 0.0042
		GIMMDA	**0.9156 ± 0.0050**	**0.9478 ± 0.0062**	**0.9218 ± 0.0105**	**0.9092 ± 0.0073**	**0.9220 ± 0.0107**

The highest value in each column is highlighted in bold, and the second-ranked value is underlined.

**Figure 3:**
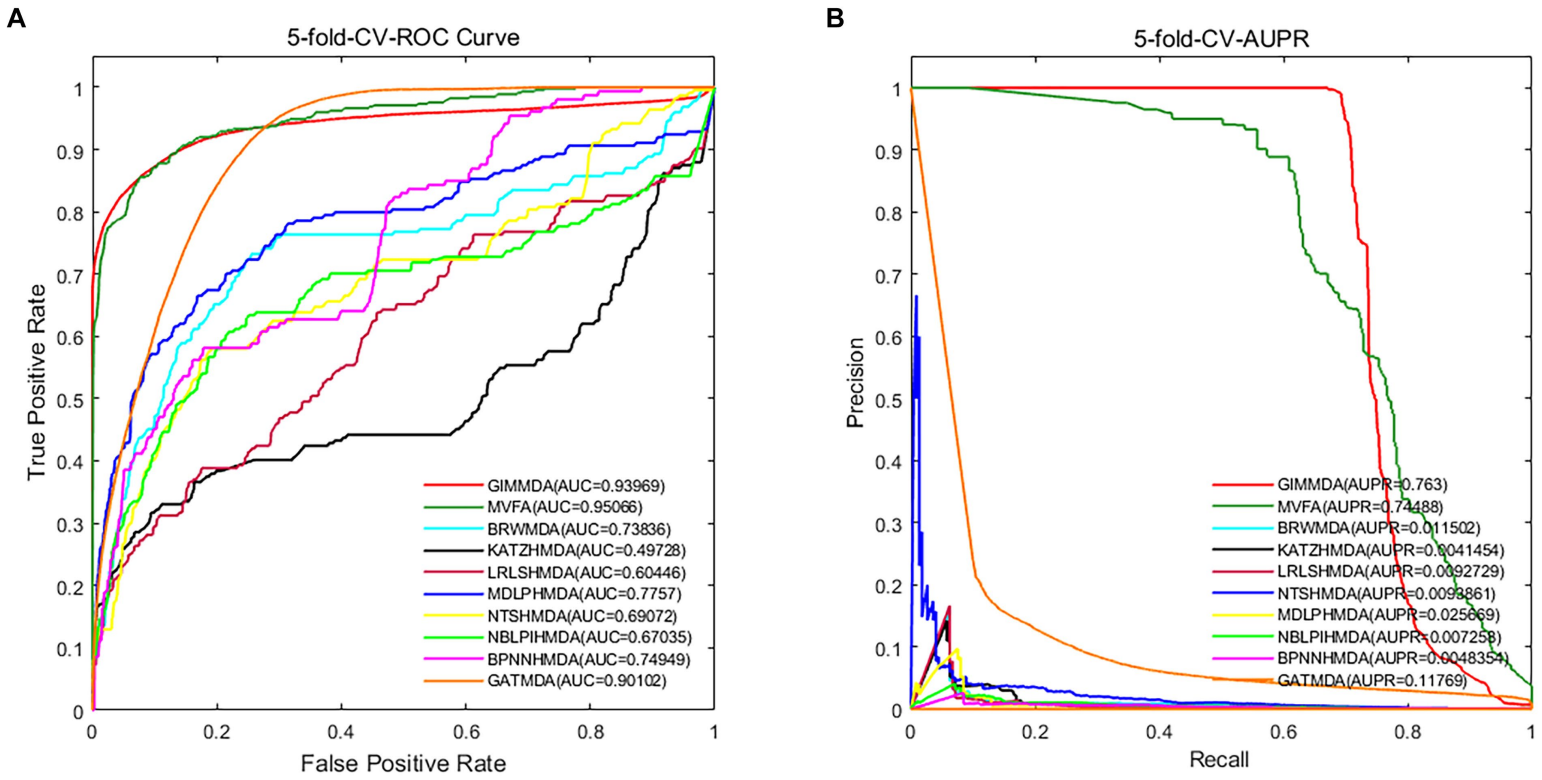
Performance comparison using the global test 5-fold CV on a multiMDA dataset for **(A)** AUROC; and **(B)** AUPRC.

In addition, we validated the robustness of these 10 methods on a multiMDA dataset. Figure 3 illustrates the area under the receiver operator characteristic (AUROC) curve and the area under the precision-recall curve (AUPRC) of the 10 MDA prediction models using the 5-fold CV on microbe–disease pairs.

Under the 5-fold CV, the GIMMDA computed a better AUC value of 0.93969 and the best AUPRC value of 0.763 among the 10 methods. These results demonstrated that the proposed GIMMDA model also achieved the best comprehensive performance compared with the other nine state-of-the-art methods on the multiMDA dataset, confirming the validity and robustness of our proposed model.

### 4.4 Ablation study

To verify whether our model can be applied to new diseases or new microbes without known associations, we performed the horizontal test for diseases and the vertical test for microbes with a 5-fold CV on the HMDAD and Disbiome datasets.

#### 4.4.1 Performance comparison under CV on diseases

As shown in Table 3, our method still outperformed other models under CV on diseases, 80% of diseases were taken as the training set and the remaining was used as the test set. The GIMMDA obtained the AUCs of 0.6763 and 0.7123, the AUC and F1 score values of the GIMMDA ranked second, and the accuracy value ranked third respectively, outperforming LRLSHMDA, NTSHMDA, BRWMDA, NBLPIHMDA, MDLPHMDA, and KATZHMDA models.

**Table 3:** Performance comparison of the 10 methods under horizontal test for disease using 5-fold CV on HMDAD and Disbiome datasets.

Datasets		Methods	AUC	F1 Score	Accuracy	Sensitivity	Specificity
HMDAD	Network-based	KATZHMDA	0.2625 ± 0.0777	0.5234 ± 0.1151	0.1649 ± 0.0371	0.3630 ± 0.1117	0.1636 ± 0.0377
		BRWMDA	0.3829 ± 0.0825	0.5769 ± 0.3827	0.3318 ± 0.1231	0.5114 ± 0.4092	0.3292 ± 0.1256
		NTSHMDA	0.4396 ± 0.1082	0.5032 ± 0.1151	0.4147 ± 0.2086	0.3434 ± 0.0966	0.4152 ± 0.2090
	Matrix factorization	NBLPIHMDA	0.3846 ± 0.1316	0.5978 ± 0.1496	0.2481 ± 0.1841	0.4430 ± 0.1602	0.2468 ± 0.1849
		MDLPHMDA	0.4498 ± 0.1240	0.6403 ± 0.1234	0.3734 ± 0.3990	0.4833 ± 0.1399	0.3713 ± 0.4017
	Machine learning	LRLSHMDA	0.3794 ± 0.1462	0.5629 ± 0.1338	0.4029 ± 0.3159	0.4032 ± 0.1266	0.4022 ± 0.3171
		GATMDA	0.4586 ± 0.0195	0.4647 ± 0.0548	0.7591 ± 0.0509	0.5050 ± 0.0520	**0.7573 ± 0.0523**
		BPNNHMDA	0.6166 ± 0.1743	**0.7129 ± 0.1619**	0.4321 ± 0.1506	0.6732 ± 0.2292	0.4289 ± 0.1522
		MVFA	**0.7664 ± 0.0512**	0.4247 ± 0.0279	**0.7793 ± 0.0125**	0.4498 ± 0.0181	0.6605 ± 0.0139
		GIMMDA	0.6763 ± 0.1085	0.6859 ± 0.2944	0.7162 ± 0.2753	**0.7459 ± 0.2193**	0.7402 ± 0.2486
Disbiome	Network-based	KATZHMDA	0.5139 ± 0.0221	0.6591 ± 0.1455	0.5243 ± 0.2764	0.5091 ± 0.1686	0.5241 ± 0.2779
		BRWMDA	0.5153 ± 0.0170	0.3883 ± 0.0282	**0.9384 ± 0.0089**	0.2413 ± 0.0220	**0.9406 ± 0.0089**
		NTSHMDA	0.5343 ± 0.0205	0.5576 ± 0.0514	0.7633 ± 0.0221	0.3883 ± 0.0503	0.7645 ± 0.0224
	Matrix factorization	NBLPIHMDA	0.5874 ± 0.0181	0.5325 ± 0.0555	0.8237 ± 0.0377	0.3648 ± 0.0536	0.8251 ± 0.0380
		MDLPHMDA	0.6900 ± 0.0044	0.7195 ± 0.0224	0.8073 ± 0.0253	0.5623 ± 0.0275	0.8081 ± 0.0255
	Machine learning	LRLSHMDA	0.6365 ± 0.0171	0.6344 ± 0.0519	0.8484 ± 0.0384	0.4666 ± 0.0570	0.8497 ± 0.0388
		GATMDA	**0.7637 ± 0.0295**	0.4679 ± 0.0089	0.7493 ± 0.0309	**0.7784 ± 0.0322**	0.7489 ± 0.0225
		BPNNHMDA	0.3118 ± 0.0133	0.1145 ± 0.0090	0.4375 ± 0.0057	0.0608 ± 0.0050	0.4387 ± 0.0057
		MVFA	0.7040 ± 0.0148	0.3395 ± 0.0396	0.4793 ± 0.0125	0.4465 ± 0.0456	0.4526 ± 0.0220
		GIMMDA	0.7123 ± 0.0212	**0.7599 ± 0.0305**	0.6856 ± 0.0353	0.6503 ± 0.0281	0.6285 ± 0.0410

The highest value in each column is highlighted in bold, and the second-ranked value is underlined.

#### 4.4.2 Performance comparison under CV on microbes

Under CV on microbes, 80% of microbes were taken as the training set and the remaining was used as the test set. Table 4 shows the performance compared with the other nine methods under CV on microbes. The GIMMDA obtained better AUC values of 0.94168 and 0.7685 compared to LRLSHMDA, NTSHMDA, BRWMDA, NBLPIHMDA, MDLPHMDA, and KATZHMDA models.

**Table 4:** Performance comparison of the 10 methods under vertical test for microbes using 5-fold CV on HMDAD and Disbiome datasets.

Datasets		Methods	AUC	F1 Score	Accuracy	Sensitivity	Specificity
HMDAD	Network-based	KATZHMDA	0.8756 ± 0.0484	0.8456 ± 0.0263	0.8641 ± 0.0418	0.7828 ± 0.0423	0.8645 ± 0.0420
		BRWMDA	0.8657 ± 0.0309	0.7985 ± 0.0493	0.9061 ± 0.0049	0.6673 ± 0.0670	**0.9438 ± 0.0053**
		NTSHMDA	0.4396 ± 0.1082	0.8430 ± 0.1151	0.8857 ± 0.0742	0.7318 ± 0.0758	0.8869 ± 0.0754
	Matrix factorization	NBLPIHMDA	0.8384 ± 0.0417	0.7968 ± 0.0496	**0.9280 ± 0.0034**	0.6651 ± 0.0705	0.9302 ± 0.0039
		MDLPHMDA	0.8019 ± 0.0288	0.8061 ± 0.0238	0.8470 ± 0.0473	0.6759 ± 0.0332	0.8484 ± 0.0478
		LRLSHMDA	0.8465 ± 0.0258	0.8267 ± 0.0499	0.8964 ± 0.0701	0.7064 ± 0.0561	0.8979 ± 0.0710
	Machine learning	GATMDA	0.9063 ± 0.0111	0.6917 ± 0.0263	0.8644 ± 0.0235	0.9091 ± 0.0214	0.8636 ± 0.0238
		BPNNHMDA	0.9057 ± 0.0112	0.8653 ± 0.0485	0.8739 ± 0.0452	0.8307 ± 0.0830	0.8744 ± 0.0462
		MVFA	0.9144 ± 0.0235	0.8112 ± 0.0193	0.9279 ± 0.0125	0.7613 ± 0.0238	0.8605 ± 0.0139
		GIMMDA	**0.9168 ± 0.0261**	**0.8918 ± 0.0331**	0.9123 ± 0.0329	**0.9286 ± 0.0540**	0.8902 ± 0.0406
Disbiome	Network-based	KATZHMDA	0.8016 ± 0.0141	0.8243 ± 0.0182	0.7915 ± 0.0218	0.7015 ± 0.0263	0.7918 ± 0.0219
		BRWMDA	0.7397 ± 0.0090	0.8152 ± 0.0142	0.6882 ± 0.0180	0.6882 ± 0.0201	0.6882 ± 0.0181
		NTSHMDA	0.6788 ± 0.0132	0.6960 ± 0.0522	0.7817 ± 0.0474	0.5361 ± 0.0606	0.7825 ± 0.0477
	Matrix factorization	NBLPIHMDA	0.6800 ± 0.0159	0.6650 ± 0.0202	0.7877 ± 0.0175	0.4984 ± 0.0227	0.7886 ± 0.0175
		MDLPHMDA	0.6304 ± 0.0114	0.6389 ± 0.0179	0.7518 ± 0.0221	0.4697 ± 0.0191	0.7527 ± 0.0221
	Machine learning	LRLSHMDA	0.7279 ± 0.0085	0.7596 ± 0.0301	0.7567 ± 0.0323	0.6134 ± 0.0399	0.7571 ± 0.0325
		GATMDA	0.8112 ± 0.0164	0.4366 ± 0.0092	0.7431 ± 0.0242	**0.8797 ± 0.0179**	0.7427 ± 0.0242
		BPNNHMDA	0.7964 ± 0.0060	0.8479 ± 0.0217	0.7046 ± 0.0292	0.7366 ± 0.0319	0.7045 ± 0.0294
		MVFA	**0.8796 ± 0.0156**	0.7536 ± 0.0039	0.7503 ± 0.0068	0.7050 ± 0.0076	**0.8647 ± 0.0211**
		GIMMDA	0.7685 ± 0.0303	**0.8841 ± 0.0920**	**0.8250 ± 0.1230**	0.3699 ± 0.1316	0.8322 ± 0.1270

The highest value in each column is highlighted in bold, and the second-ranked value is underlined.

#### 4.4.3 Performance comparison under different similarity fusion methods

To verify the effectiveness of our proposed similarity fusion method, we compared the model performance while using the proposed strategy and the other 3 similarity fusion methods.

Proposed strategy (SNF-LNF): the proposed similarity fusion strategy was explained in detail in Section 2.4. We set the optimal parameter μ as 0.5.

Compared strategies:

Strategy 1 (LNF-LNF): for the same attributes within network topology or biological function information and different attributes between network topology and biological function information, we all selected the LNF method to average the similarity matrixes.
Strategy 2 (SNF-SNF): in this strategy, we adopted the SNF method to fusion all similarity matrixes constructed from network topology and biological function information.
Strategy 3 (LNF-SNF): it took the opposite method to our proposed strategy, LNF was applied to fuse the same attributes and SNF was used for similarity fusion between different attributes.

**Figure 4:**
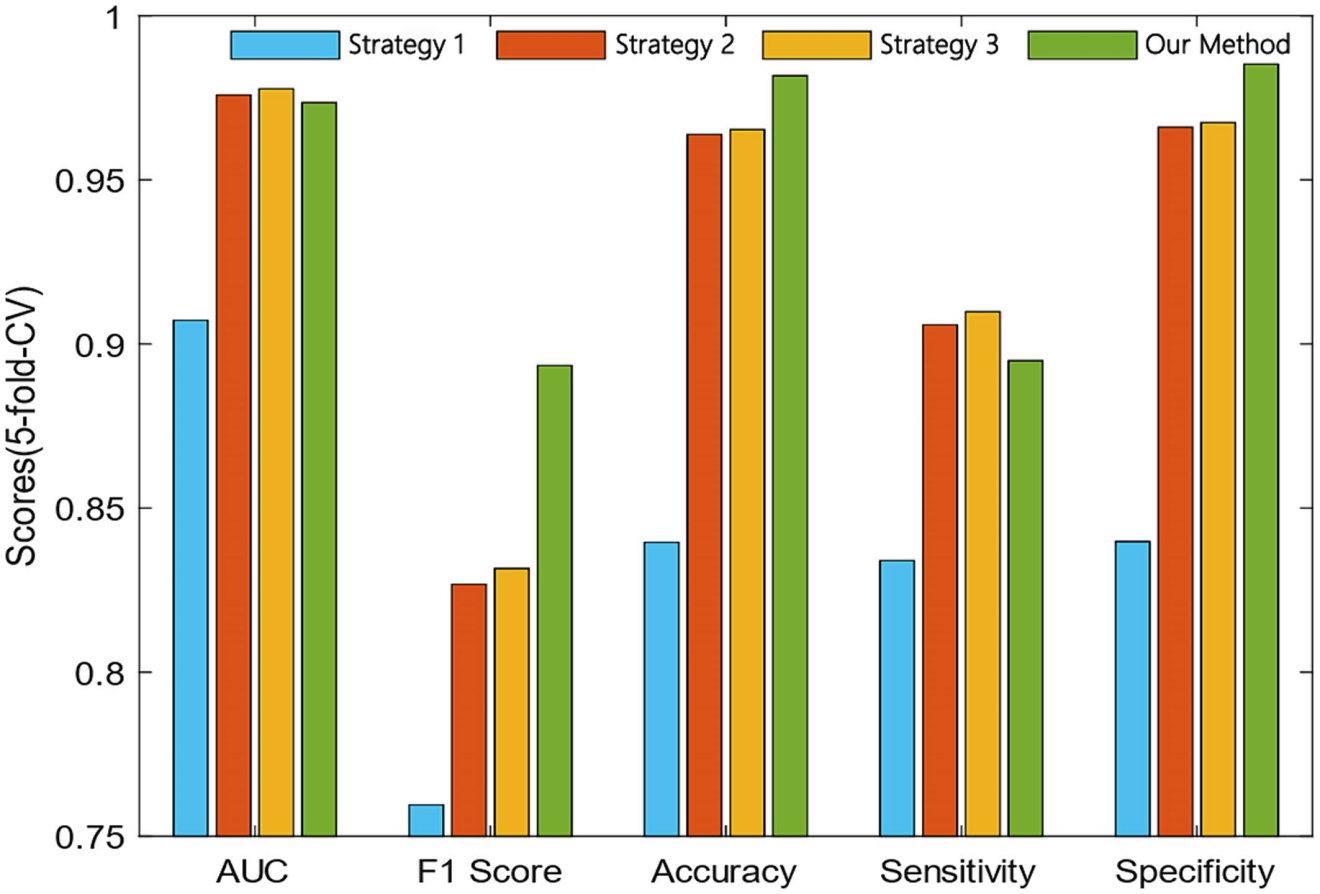
Comparative analysis of the proposed and compared similarity fusion methods.

The comparison results of our proposed method and the other three strategies are shown in Figure 4. We can observe that the AUC of strategy 3 was 0.42% higher than our method. However, the F1 score and accuracy of our method were 6.18 and 1.64% higher than the strategy 3.

### 4.5 Case study

To further validate the predictive performance of the proposed GIMMDA, we selected two common diseases, i.e., asthma and obesity as case studies on the multiMDA dataset. After excluding known related microbes for these two diseases, the top 20 microbe candidates were selected based on the ranking scores for validation by searching previous publications. The results showed that 19 and 14 of the top 20 bacteria linked to asthma and obesity were confirmed by previous literature studies. Tables 5, 6 present the findings of the literature validation of the top 20 predicted microbes linked to obesity and asthma.

**Table 5:** Prediction results of the top 20 asthma-associated microbes.

Rank	Microbe	Evidence	Rank	Microbe	Evidence
1	Proteobacteria	PMID:35685081	11	*Clostridium coccoides*	PMID:21477358
2	*Helicobacter pylori*	PMID:28389738	12	Bacteroides	PMID:18822123
3	Bacteroidetes	PMID:25306600	13	Eubacterium	PMID:32506557
4	Prevotella	PMID:34612663	14	Pseudomonas	PMID:13268970
5	Haemophilus	PMID:35904980	15	Lachnospiraceae	PMID:31958431
6	Comamonadaceae	PMID:21194740	16	Porphyromonadaceae	PMID:28947029
7	Oxalobacteraceae	PMID:21194740	17	Bacteroidaceae	PMID:28947029
8	Sphingomonadaceae	PMID:21194740	18	*Fusobacterium nucleatum*	PMID:28486933
9	Staphylococcus	PMID:5601136	19	Actinobacteria	PMID:23265859
10	*Bacteroides vulgatus*	Unconfirmed	20	Veillonella	PMID:26424567

The second column records 1–10 related microbes, and the fifth column records 11–20 related microbes.

**Table 6:** Prediction results of the top 20 obesity-associated microbes.

Rank	Microbe	Evidence	Rank	Microbe	Evidence
1	*Staphylococcus aureus*	PMID:29026443	11	Lysobacter	Unconfirmed
2	Firmicutes	PMID:35682774	12	Xanthomonas	PMID:30146618
3	Alcaligenaceae	Unconfirmed	13	Rickettsiales	Unconfirmed
4	Coriobacteriaceae	PMID:29030493	14	*Streptococcus mitis*	PMID:35906254
5	Erysipelotrichaceae	PMID:27304513	15	*Shigella dysenteriae*	Unconfirmed
6	*Methanobrevibacter smithii*	PMID:23459324	16	*Enterobacter aerogenes*	PMID:20736424
7	Bacteroidetes	PMID:35682774	17	*Enterobacter hormaechei*	PMID:21572120
8	Prevotellaceae	PMID:19164560	18	*Staphylococcus epidermidis*	PMID:7776298
9	*Fusobacterium nucleatum*	PMID:27717180	19	*Dietzia maris*	Unconfirmed
10	Verrucomicrobiaceae	PMID:32466962	20	Comamonadaceae	Unconfirmed

The second column records 1–10 related microbes, and the fifth column records 11–20 related microbes.

Asthma is a common chronic inflammatory disease of the airways that is estimated to affect more than 300 million people worldwide over the life course (Varkonyi-Sepp et al., 2022). A large literature has reported that the development of asthma is closely related to genes, the environment, and specific microbes in the gut or respiratory tract (Frei et al., 2022). In our predictions, Proteobacteria, *Helicobacter pylori*, Bacteroidetes, and Prevotella were the most relevant influencing factors for asthma in the top 20 score list. Zhang et al. found that the relative abundance of Proteobacteria was significantly enrichment and lower levels of Bacteroidota (synonym Bacteroidetes) in the asthma patients group compared to that in the control group (Zhang et al., 2022). Wang D. et al. (2022) demonstrated a significant inverse correlation between *Helicobacter pylori* infection and asthma. Clinical studies have shown that patients with asthma have reduced numbers of Prevotella compared to healthy individuals (Gu et al., 2023). Except for microbes validated by literature, *Bacteroides vulgatus* has no direct evidence of an association with asthma. There was a report that *Bacteroides vulgatus* appeared to increase in relative abundance in depression patients (Liang et al., 2022), where depression was a comorbidity of asthma (Tamayo et al., 2024). In other words, conventional biological experiments will further verify the significant role that microbes play in asthma, for which there is currently no direct evidence.

Obesity is another major global health problem determined by genetics and environment, and its incidence is increasing every year (Liu B. N. et al., 2021). Extensive studies have shown that gut microbiota is an important factor in the development of metabolic diseases such as obesity. Megur, Daliri, Baltriukiene, and Burokas (Megur et al., 2022) detected that obesity is associated with an increase in the number of Firmicutes and a decrease in the diversity of Bacteroidetes, where they were all in the top 20 score list. In addition, Alcaligenaceae in the top 20 score list was rarely reported about obesity, but Ishaq, Mohammad, Hussain, Parveen, Shirazi, Fan, Shahzad, Hayat, Li, Ihsan, Muhammad, Usman, Zhang, Yuan, Ullah, Paiva-Santos, and Xu (Ishaq et al., 2022) found that the abundance of Alcaligenaceae was significantly reduced in patients with thyroid cancer compared with healthy individuals, where thyroid cancer had an association with obesity (Rahman et al., 2020). Another less-reported microbe related to obesity was Lysobacter. However, there was evidence that Lysobacter can produce cellulase, which has the potential use in preventing obesity and restoring intestinal homeostasis in obese individuals (Fu et al., 2021). In the future, microbes in our prediction results that have not been validated in literature may also serve as novel biomarkers for obesity. In conclusion, the prediction results illustrated that our proposed method can accurately and reliably predict the microbe–disease, which can contribute to the diagnosis, treatment, and prevention.

## 5 Discussion

In recent years, researchers have paid more attention to the associations between microbes and diseases. However, biological experiments are expensive and inefficient for screening the microbe–disease associations. Therefore, more and more computational methods are used to predict potential microbe–disease interactions. In addition, the primary challenges of computational models are the reliability of the data, the richness of prior knowledge, and the prediction accuracy of the model.

In this study, we developed an end-to-end deep learning framework GIMMDA based on graph autoencoders and the inductive matrix completion with multiple similarities fusion. First, we integrated and screened a new dataset (multiMDA) from six diverse association databases. Second, we fused the known microbe–disease association and different priori knowledge by SNF and LNF methods to the microbe similarity network and disease similarity network, respectively. Third, we learned the node feature representations by the information from node neighbors and itself based on graph convolutional networks. Finally, we adopted collaborative optimization of the loss to obtain the final microbe–disease association prediction scores. In addition, comparative experiments with nine other models and case studies of two diseases showed that our proposed GIMMDA model achieved superior predictive performance, excellent reliability, and broad application.

However, our proposed model still has some limitations that need to be improved in the future. First, although we integrated six databases, the known associations are still sparse compared with the entire association space, which affected the prediction performance. Self-supervised learning provides a solution to address association sparseness. Second, the proposed GIMMDA model still lacked prior knowledge and needed to be further explored, such as the abundance, metabolism, gene sequence information of microbes, drugs, and metabolites information of diseases. In the future, we will make further improvements to overcome these shortcomings.

## Data Availability

The original contributions presented in the study are publicly available. This data can be found here: [https://ftp.ncbi.nlm.nih.gov/blast/db/].

## Footnotes

^1^http://www.nlm.nih.gov/
